# Effectiveness of a forgiveness-based intervention to promote post-traumatic growth in hemodialysis patients: an experimental controlled study

**DOI:** 10.3389/fpsyg.2025.1680748

**Published:** 2025-10-20

**Authors:** Qin Xie, Yue Zhang, Yansheng Ye, Hong Wang, Dan Luo, Changwei Mu

**Affiliations:** ^1^School of Nursing, Chengdu University, Chengdu City, Sichuan Province, China; ^2^School of Nursing, Dali University, Dali City, Yunnan Province, China; ^3^Diagnosis and Treatment Center of Integrated Chinese and Western Medicine, Chongqing General Hospital, Chongqing University, Chongqing, China; ^4^The People's Hospital of Yuxi City, Yuxi, Yunnan, China; ^5^Blood Purification Center, The Affiliated Hospital of Chengdu University, Chengdu, Sichuan Province, China

**Keywords:** forgiveness, post-traumatic growth, maintenance hemodialysis, psychological intervention, depression, anxiety

## Abstract

**Background:**

Post-Traumatic Growth (PTG) is a key indicator of psychological wellbeing and quality of life in patients undergoing Maintenance Hemodialysis (MHD). Forgiveness, as a positive psychological process, involves transforming negative responses into adaptive ones after trauma and may facilitate PTG by reducing resentment and promoting emotional recovery. However, empirical evidence on the effectiveness of forgiveness-based interventions in enhancing PTG remains limited, particularly among hemodialysis patients.

**Objective:**

This study aimed to develop a forgiveness-based intervention program for post-traumatic growth in patients undergoing MHD and to evaluate its effectiveness.

**Methods:**

This study was conducted in a tertiary hospital in Yunnan Province with 78 maintenance hemodialysis patients assigned to an intervention group (*n* = 39) and a control group (*n* = 39). The intervention group received an 8 week nurse-delivered forgiveness-based psychological program alongside routine care, while the control group received routine care only. Post-traumatic growth, forgiveness, coping style, anxiety, depression, heart rate, and blood pressure were assessed at baseline, post-intervention, and 3 month follow-up. Data analysis was performed using descriptive statistics, independent *t* tests, chi-square or Fisher's exact tests, repeated-measures ANOVA, and paired *t* tests. Statistical significance was set at *p* < 0.05.

**Results:**

Both groups were comparable at baseline across all outcome measures. Post-traumatic growth significantly increased in the intervention group compared to the control group at the end of the intervention (T2: *p* < 0.001) and at three-month follow-up (T3: *p* < 0.001). Forgiveness scores were also significantly higher in the intervention group at both T2 and T3 (*p* < 0.001). Positive coping showed significant improvement at both time points (*p* < 0.001), while negative coping, anxiety, and depression scores were significantly reduced (all *p* < 0.01). No significant differences were found between groups in heart rate or blood pressure at any time point.

**Conclusions:**

The forgiveness-based intervention program showed beneficial effects on post-traumatic growth, forgiveness, and emotional adjustment in patients receiving maintenance hemodialysis. As a low-cost approach, it has the potential to be incorporated into routine dialysis care to help promote post-traumatic growth in patients receiving maintenance hemodialysis.

**Clinical trial registration:**

https://www.chictr.org.cn/showproj.aspx?proj=184556, identifier Chinese Clinical Trial Registry (ChiCTR2200066914).

## Introduction

End-Stage Renal Disease (ESRD), the final stage of Chronic Kidney Disease (CKD), is defined by irreversible renal failure, necessitating dialysis or kidney transplantation for survival (Francis et al., 2024; Chen et al., 2025). The latest USRDS 2024 data show that both the prevalence and number of ESRD cases have increased markedly in recent years (United States Renal Data System, 2024). Globally, approximately 2.6 million people received Kidney Replacement Therapy (KRT) in 2010, and this number is projected to increase to 5.4 million by 2030 (Thurlow et al., 2021). In China, the prevalence of treated ESRD reached 697.4 per million population (pmp) in 2022 (Chen et al., 2025). Among the available modalities of KRT, hemodialysis is the most commonly used worldwide. Hemodialysis (HD) accounts for approximately 69% of all KRT and 89% of all dialysis treatments (Bello et al., 2022). HD is an extracorporeal blood purification therapy, usually performed three times weekly for several hours per session, requiring long-term vascular access such as an arteriovenous fistula, graft, or catheter (Bello et al., 2022; Lok et al., 2024). The global hemodialysis population exceeded 2.5 million in 2020 and is projected to rise to 5.4 million by 2030 (Anon, 2020; Bello et al., 2022). In China, Maintenance Hemodialysis (MHD) is the predominant treatment for ESRD, covering about 86.7% of patients (Wang et al., 2021). As of 2022, more than 844,000 individuals were receiving MHD, making China the country with the largest hemodialysis population worldwide (Chen et al., 2025).

Patients receiving MHD often face substantial challenges, including financial strain, lifestyle limitations, and dialysis-related complications such as pruritus, fatigue, and malnutrition (Chaiviboontham et al., 2020; Jha et al., 2023). Compared with other renal replacement therapies, MHD imposes a heavier treatment burden and is associated with lower quality of life (Fradelos et al., 2021; Guerra et al., 2021). The chronic nature of End-Stage Renal Disease (ESRD) and the continuous demands of dialysis are often experienced as traumatic, leading to psychological distress such as anxiety, depression, demoralization, and diminished life satisfaction (Fradelos et al., 2021; Guerra et al., 2021; Shdaifat et al., 2024). While many patients experience these adverse effects, some demonstrate positive psychological adaptation to chronic illness (Zegarow et al., 2020; Yang et al., 2024), including the potential to develop PTG.

Post-Traumatic Growth (PTG), first conceptualized by Tedeschi and Calhoun in 1996, refers to positive psychological changes in self-perception, interpersonal relationships, and life meaning following traumatic experiences (Tedeschi and Calhoun, 1996, 2004). Evidence suggests that MHD patients may experience PTG, reflected in life re-evaluation, enhanced appreciation of daily life, and improved self-awareness over the course of long-term treatment (Tedeschi and Calhoun, 2004; Cui et al., 2017; Zhang and Cao, 2024; Arpaci and Tanriverdi, 2025). Studies have further indicated that promoting PTG may contribute to improved psychological outcomes and better treatment adherence (Ha et al., 2019; Li et al., 2020). However, empirical research directly exploring the relationship between forgiveness and PTG in patients receiving hemodialysis remains limited.

This study developed a forgiveness-based psychological intervention program to promote PTG in patients receiving MHD. The intervention was based on the Enright Forgiveness Process Model, one of the most widely applied frameworks in forgiveness research (Freedman and Enright, 1996; Kurtines et al., 2013). Forgiveness interventions have demonstrated effectiveness across diverse populations, including trauma-exposed students (Vassilopoulos et al., 2020), bereaved parents (Záhorcová et al., 2023), individuals with alcohol dependence (Scherer et al., 2011), and forensic psychiatric patients. For example, Ha et al. (2019) found that a four-session forgiveness writing intervention significantly reduced PTSD symptoms, shame, depression, and maladaptive coping, while promoting PTG in survivors of sexual abuse. However, evidence regarding the use of forgiveness-based interventions in patients with MHD remains scarce, despite their high levels of symptom burden, psychosocial distress, and reduced quality of life. Addressing this gap is clinically important, as it may provide nurses with a feasible psychological strategy to support this vulnerable population. Based on this evidence, we hypothesized that participants receiving the forgiveness-based program would exhibit greater improvements in PTG and related psychological outcomes compared to those receiving standard care. Accordingly, the aim of this study was to evaluate the effectiveness of the forgiveness-based intervention in enhancing PTG, increasing forgiveness, improving coping strategies, and reducing anxiety and depression among patients undergoing MHD.

## Methods

### Study design and participants

The study was conducted at a tertiary hospital in Yunnan Province, China. Patients were recruited from the Blood Purification Center of the Department of Nephrology between June and December 2023.Inclusion criteria were: (1) age ≥18 years; (2) ability to communicate effectively; (3) undergoing Maintenance Hemodialysis (MHD) three times per week for at least 3 months; and (4) provision of written informed consent. Exclusion criteria were: (1) severe physical comorbidities, such as malignancy; (2) history of psychiatric disorders; and (3) current or recent (within the past 3 months) psychological counseling, psychotherapy, or psychiatric medication. Elimination criteria were: (1) voluntary withdrawal due to intolerance of the intervention; (2) failure to complete scheduled follow-up assessments; and (3) receipt of kidney transplantation or other major surgery during the study period. To minimize potential bias associated with differences in treatment schedules, patients were stratified according to their dialysis shifts: those attending sessions on Monday, Wednesday, and Friday were categorized as Group A, while those dialyzing on Tuesday, Thursday, and Saturday were classified as Group B. Within each stratum, participants were randomly allocated to either the intervention or control group using a computer-generated random number table. A total of 39 participants were enrolled in each group, all of whom completed the baseline assessments and remained in the study throughout the intervention and follow-up periods.

**Figure 1:**
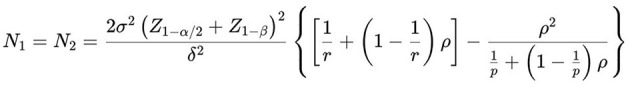
Sample size calculation formula based on repeated measures ANOVA.

The sample size was estimated using the repeated measures ANOVA formula (Sakpal, 2010) (Figure 1), with parameter values derived from a previous intervention study targeting PTG in patients receiving MHD (Liu, 2020). Accounting for a projected 20% attrition rate, the final required sample size was calculated to be 39 participants per group.

## Intervention

### Control group

Participants in the control group continued to receive routine clinical care without the forgiveness-based psychological intervention.

### Forgiveness-based intervention

The intervention group received standard care in addition to an 8 week forgiveness-based psychological intervention designed to promote posttraumatic growth (PTG).

The intervention group received standard care plus an 8 week forgiveness-based psychological program designed to promote Posttraumatic Growth (PTG). The program consisted of weekly individual sessions (60–90 mins) delivered during dialysis by trained researchers, following Enright's forgiveness process model with four stages: uncovering, decision, work, and deepening. Sessions combined psychoeducation, cognitive-behavioral strategies, guided imagery, role-play, and self-care training (e.g., vascular access care, weight management, dietary guidance). Additional support was provided via telephone and WeChat. Each session included a review, structured activities, and follow-up assignments to reinforce learning and adherence. Details of weekly objectives, contents, and methods are presented in Table 1.

**Table 1:** Overview of the forgiveness-based intervention to Promote Post-Traumatic Growth (PTG).

**Week**	**Theme**	**Objectives**	**Content**	**Methods**
1–2	Experiencing injury	Elicit patients' dialysis-related distress and emotional responses. Provide basic education on disease and coping. Identify the risks of negative emotions.	Narrative exploration of dialysis experiences. Education on dialysis principles and psychosocial stressors. Relaxation training.	Face-to-face interviews, lecture, demonstration, practice.
3–4	Decision to forgive	Promote cognitive reframing of dialysis experience. Introduce forgiveness concepts and coping strategies. Address maladaptive beliefs.	ABC theory of emotion. Analysis of cause–effect patterns in dialysis-related distress. Guided imagery and reflection.	Group discussion, case-based teaching, visualization.
5–6	Practicing forgiveness	Foster patients' willingness to forgive self and others. Deepen understanding of PTG. Recognize benefits of forgiveness.	Storytelling and guided empathy exercises. Reflection on personal and vicarious forgiveness cases. Cognitive-behavioral strategies for emotional regulation.	Visual materials, role-play, health coaching.
7–8	Deepening forgiveness	Consolidate forgiving attitudes and PTG-related thinking. Explore positive reinterpretation of suffering. Build future-oriented goals.	Revisiting key lessons from prior sessions. Physical activity and fluid/diet control education as self-regulation practice. Group reflection on life meaning.	Homework assignments, motivational messaging, WeChat support.

### Outcomes and measurements

Participants were assessed at three time points: baseline (T1, pre-intervention), post-intervention (T2), and 3 month follow-up (T3). The primary outcome was posttraumatic growth (PTG). Secondary outcomes included forgiveness, coping style, anxiety, depression, and physiological indicators (heart rate and blood pressure). All assessments were conducted using paper-based questionnaires administered during dialysis sessions.

### Socio-demographic and hemodialysis-related characteristics

A researcher-developed general information questionnaire was used to collect participants' demographic and dialysis-related data at baseline. Demographic variables included age, sex, marital status, education level, employment status, monthly household income, and religious belief. Dialysis-related information, such as the primary cause of end-stage renal disease (ESRD), dialysis modality, duration of dialysis, vascular access type, and interdialytic weight gain, was obtained from both patient self-report and medical records.

### Primary outcome

PTG was evaluated using the Chinese version of the Posttraumatic Growth Inventory (C-PTGI) (Tedeschi and Calhoun, 1996). The scale comprises 20 items distributed across five dimensions: relating to others, new possibilities, appreciation of life, personal strength, and self-transformation. Respondents indicate the degree of positive change they experienced after a traumatic event using a 6-point Likert scale (0 = not at all, 5 = very much). Higher overall scores represent greater levels of PTG, with the total ranging from 0 to 100. The Chinese version of the C-PTGI has demonstrated good internal consistency in prior validation studies, with a reported Cronbach's α of 0.874(Wang et al., 2011). In the present study, this validated version was adopted without recalculating internal reliability.

### Secondary outcome

Forgiveness was measured via the revised Heartland Forgiveness Scale (HFS) (Thompson et al., 2005), which includes 24 items covering the dimensions of forgiveness of self and others. Each item is rated on a 7-point scale (1 = strongly disagree, 7 = strongly agree), providing a total score from 24 to 168. This instrument captures trait-level forgiveness tendencies and demonstrated good internal consistency in the current sample (Cronbach's α = 0.78) (Wang, 2006).

Anxiety and depression symptoms were assessed using the Hospital Anxiety and Depression Scale (HADS) (Zigmond and Snaith, 1983), a well-established tool consisting of 14 items −7 for anxiety and 7 for depression. Participants responded on a 4-point scale indicating the frequency or severity of symptoms experienced during the past week. Subscale scores range from 0 to 21, with higher values reflecting more severe emotional distress. A cut-off score of 9 or above suggests probable clinical relevance. Cronbach's alphas in this sample were 0.76 (anxiety) and 0.79 (depression), respectively (Zheng et al., 2003).

Coping style was captured using the Simplified Coping Style Questionnaire (Xie, 1998). The scale contains 20 items that are divided into two separate subscales: positive coping (12 items) and negative coping (8 items). Participants rated their coping behavior on a 4-point Likert scale ranging from 0 (never) to 3 (often). Scores are calculated separately for each subscale, with higher scores reflecting more frequent use of that coping strategy. Internal consistency for the questionnaire was high, with an overall Cronbach's alpha of 0.90 (Xie, 1998).

Physiological indicators, including heart rate and blood pressure, were measured prior to dialysis sessions. Participants were instructed to rest for 15 mins in a quiet environment before measurement. A validated automatic medical device (OMRON HBP-9030) was used to obtain systolic and diastolic pressure as well as heart rate, providing objective data on autonomic function and cardiovascular status.

### Data analysis

Descriptive analyses were performed to summarize the characteristics of the total sample and each group. Categorical variables were presented as frequencies and percentages, while continuous variables were expressed as means ± standard deviations (SD) for normally distributed data or medians with interquartile ranges (IQR) for skewed data. Baseline differences between groups were assessed using independent samples *t*-tests or one-way analysis of variance (ANOVA) for normally distributed continuous variables, and Mann–Whitney *U* tests for non-normally distributed variables. Categorical variables were compared using chi-square tests or Fisher's exact tests, as appropriate. To evaluate the effects of the intervention, both within-group and between-group comparisons were conducted. Paired *t*-tests were used for normally distributed within-group comparisons, and independent *t*-tests for between-group comparisons. For non-normally distributed data, Wilcoxon signed-rank tests and Mann–Whitney *U* tests were applied. Repeated-measures ANOVA was used to assess time-by-group interaction effects when assumptions were met. All analyses were conducted using IBM SPSS Statistics, version 25.0, with a significance level set at *p* < 0.05.

### Ethics

This study was approved by the Hospital Ethics Committee (Approval No. 2022kmykdx6f171) and was registered in the Chinese Clinical Trial Registry (Registration No. ChiCTR2200066914).

## Results

### Baseline comparisons

**Figure 2:**
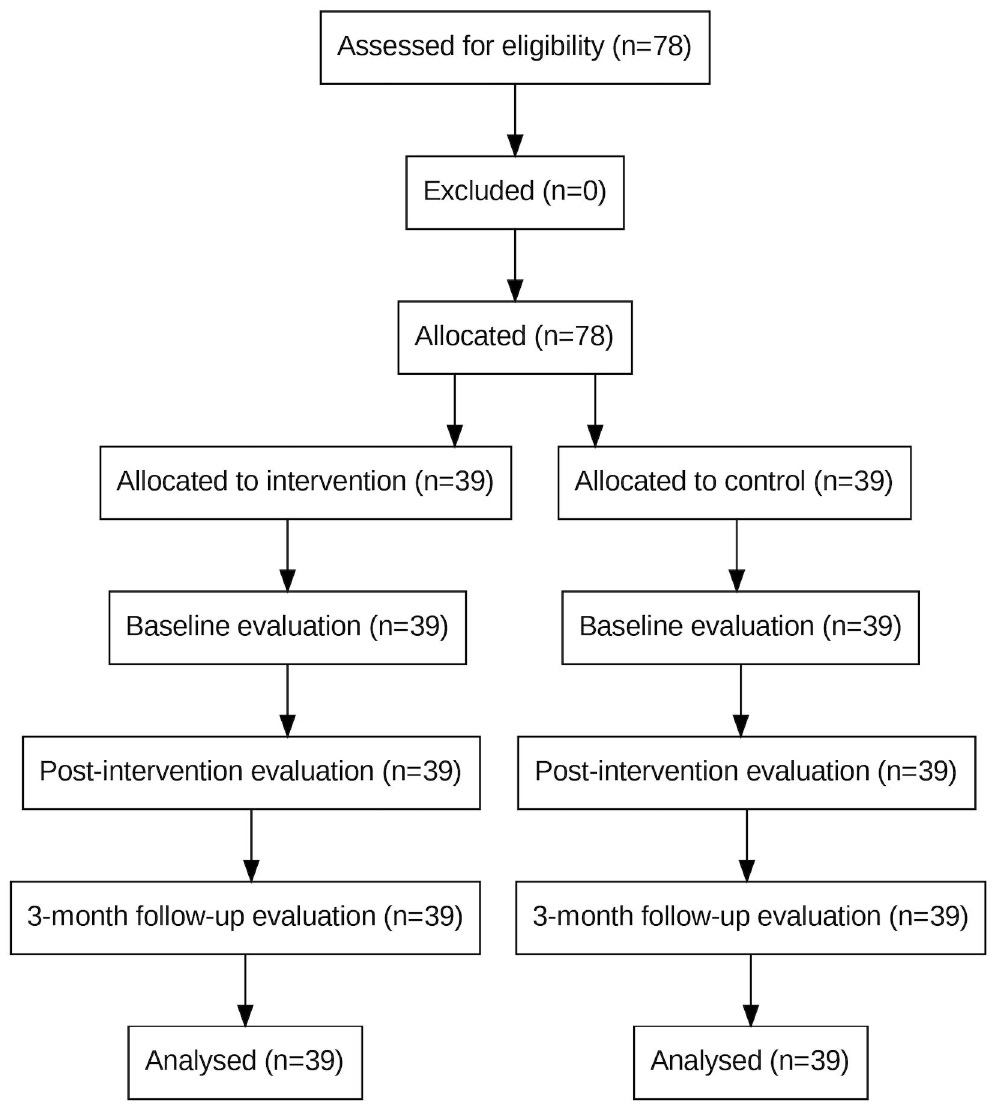
Flow chart of the sample and phases of the study.

A total of 78 patients receiving maintenance hemodialysis were enrolled between June and December 2023. After screening for eligibility, 39 were allocated to the intervention group and 39 to the control group. All participants completed the baseline, post-intervention, and 3 month follow-up assessments. No participants withdrew or were lost to follow-up. Baseline characteristics did not differ significantly between groups (Table 1). The flow of participants through the study is shown in the CONSORT diagram (Figure 2).

Table 2 presents the sociodemographic and clinical characteristics of the participants. No statistically significant differences were observed between the intervention and control groups across all demographic or clinical variables (all *P* > 0.05). Table 3 displays the baseline scores of the two groups on all primary and secondary outcome measures. No significant differences were found in PTG, forgiveness, coping style, anxiety, depression, heart rate, or blood pressure between the two groups at baseline (all *P* > 0.05), indicating comparability prior to the intervention.

**Table 2:** Baseline socio-demographic and clinical characteristics of participants (*N* = 78).

**Variables**	**Total (*N* = 78)**	**Intervention group (*n* = 39)**	**Control group (*n* = 39)**	**Test statistic**	***p*-Value**
Age, years (Median, IQR)	54.00 (43.75, 59.00)	51.00 (42.00, 58.00)	56.00 (46.00, 60.00)	1.651	0.099^b^
**Sex, n (%)**				1.285	0.257
Male	37 (47.44)	16 (41.03)	21 (53.85)		
Female	41 (52.56)	23 (58.97)	18 (46.15)		
**Ethnicity, n (%)**				1.258	0.262
Han	62 (79.49)	29 (74.36)	33 (84.62)		
Other	16 (20.51)	10 (25.64)	6 (15.38)		
**Residence, n (%)**				0.891	0.345
Urban	50 (64.10)	27 (69.23)	23 (58.97)		
Rural	28 (35.90)	12 (30.77)	16 (41.03)		
**Education, n (%)**				-	0.327^a^
Junior high or below	55 (70.51)	27 (69.23)	28 (71.79)		
Senior high	7 (8.98)	4 (10.26)	3 (7.69)		
College or above	16 (20.51)	8 (20.51)	8 (20.51)		
**Religious belief, n (%)**				-	1.000^a^
Yes	7 (8.98)	3 (7.69)	4 (10.26)		
No	71 (91.02)	36 (92.31)	35 (89.74)		
**Marital status, n (%)**				-	1.000^a^
Married	72 (92.31)	36 (92.31)	36 (92.31)		
Other	6 (7.69)	3 (7.69)	3 (7.69)		
**Employment status, n (%)**				1.576	0.209
Unemployed/Retired	66 (84.62)	31 (79.49)	35 (89.74)		
Employed	12 (15.38)	8 (20.51)	4 (10.26)		
**Occupation, n (%)**				-	0.647^a^
Staff/Clerk	25 (32.05)	10 (25.64)	15 (38.46)		
Worker	8 (10.25)	5 (12.82)	3 (7.69)		
Farmer	23 (29.49)	12 (30.77)	11 (28.21)		
Other	22 (28.21)	12 (30.77)	10 (25.64)		
**Average monthly income, n (%)**				0.315	0.575
< 5000 yuan	62 (79.49)	30 (76.92)	32 (82.05)		
5000–10000 yuan	16 (20.51)	9 (23.08)	7 (17.95)		
Medical payment method, n (%)				0.466	0.495
Employment insurance	35 (44.87)	16 (41.03)	19 (48.72)		
Urban/rural insurance	43 (55.13)	23 (58.97)	20 (51.28)		
Dialysis duration, months	36.50 (15.75, 85.25)	30.00 (12.00, 76.00)	49.00 (20, 108)	1.435	0.151^b^
Interdialytic weight gain (kg)	2.65 ± 0.89	2.58 ± 0.80	2.72 ± 0.90	−0.701	0.486
**Primary etiology, n (%)**				-	0.751^a^
Chronic glomerulonephritis	43 (55.13)	23 (58.97)	20 (51.28)		
Diabetic nephropathy	17 (21.80)	9 (23.08)	8 (20.51)		
Hypertensive nephropathy	5 (6.41)	2 (5.13)	3 (7.69)		
Gouty nephropathy	1 (1.28)	1 (2.56)	0 (0.00)		
Polycystic kidney disease	2 (2.56)	0 (0.00)	2 (5.13)		
Others	9 (11.54)	5 (12.82)	4 (10.27)		
**Dialysis access, n (%)**				-	0.201^a^
Autogenous AV fistula	70 (89.74)	37 (94.87)	33 (84.62)		
CVC	3 (3.85)	0 (0.00)	3 (7.69)		
Transplanted blood vessel	5 (6.41)	2 (5.13)	3 (7.69)		
**Dialysis complication number**				-	0.690^a^
0	2 (2.56)	2 (5.13)	0 (0.00)		
1	6 (7.69)	3 (7.69)	3 (7.69)		
2	3 (3.85)	1 (2.56)	2 (5.13)		
≥3	68 (87.18)	33 (84.61)	35 (89.75)		

Data are presented as mean ± SD or median (IQR) for continuous variables and n (%) for categorical variables. Independent t-test was used for normally distributed continuous variables, Mann–Whitney U test (^b^) for non-normally distributed variables, and chi-square test (χ^2^) or Fisher's exact test (^a^) for categorical variables.

**Table 3:** Baseline comparison of posttraumatic growth and related variables between groups (*N* = 78).

**Variables**	**Total (*N* = 78) Mean ±SD**	**Intervention group (*n* = 39) Mean ±SD**	**Control group (*n* = 39) Mean ±SD**	***T* value**	***P* value**
Posttraumatic growth	50.06 ± 8.71	49.97 ± 10.10	50.15 ± 7.18	−0.090	0.928
Forgiveness	119.94 ± 16.99	117.46 ± 18.76	122.41 ± 14.84	−1.292	0.200
**Coping style**					
Positive coping	16.90 ± 4.09	16.92 ± 4.28	16.87 ± 3.95	0.055	0.956
Negative coping	9.94 ± 3.34	10.38 ± 4.00	9.49 ± 2.47	1.191	0.238
**Anxiety and depression**					
Anxiety	4.29 ± 2.74	4.79 ± 3.36	3.79 ± 1.84	1.632	0.108
Depression	3.71 ± 2.71	3.85 ± 3.04	3.59 ± 2.37	0.415	0.679
Heart rate (beats/min)	83.50 ± 11.70	83.33 ± 11.33	83.67 ± 12.20	−0.125	0.901
Systolic BP (mmHg)	138.15 ± 16.28	137.33 ± 16.02	138.97 ± 16.71	−0.443	0.659
Diastolic BP (mmHg)	76.01 ± 11.39	75.74 ± 10.99	76.28 ± 11.92	−0.207	0.836

Data are presented as mean ± standard deviation (SD).

#### Posttraumatic growth

**Figure 3:**
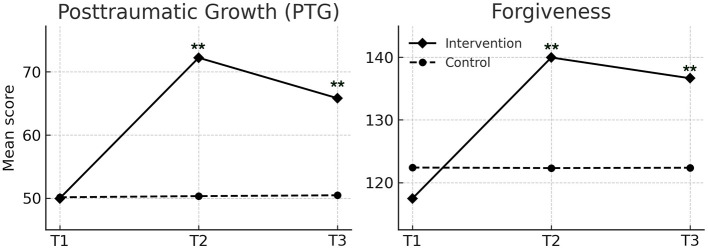
Changes in posttraumatic growth (PTG) and forgiveness scores over time in the intervention and control groups. Values are presented as means. T1, T2, and T3 indicate baseline, post-intervention, and 3-month follow-up, respectively. Repeated-measures ANOVA showed significant effects of group, time, and group × time interaction for both PTG (*p* < 0.001) and forgiveness (*p* < 0.01). ^**^ indicates *p* < 0.01 for between-group comparisons at the same time point.

At the end of the intervention (T2), the intervention group had significantly higher PTG scores (*M* = 72.23, *SD* = 11.88) compared to the control group (*M* = 50.33, *SD* = 6.08, *P* < 0.001). At the 3 month follow-up (T3), the intervention group maintained significantly higher PTG scores (*M* = 65.82, *SD* = 10.66) than the control group (*M* = 50.46, *SD* = 6.45, *P* < 0.001) (Figure 3). No significant differences were observed at baseline.

#### Forgiveness

Forgiveness scores were significantly higher in the intervention group at T2 (*M* = 139.95, *SD* = 14.81) and T3 (*M* = 136.64, *SD* = 12.66) compared to the control group (T2: *M* = 122.33, *SD* = 14.49; T3: *M* = 122.38, *SD* = 14.25; both *P* < 0.001) (Figure 3). No differences were found at baseline.

#### Coping style

**Figure 4:**
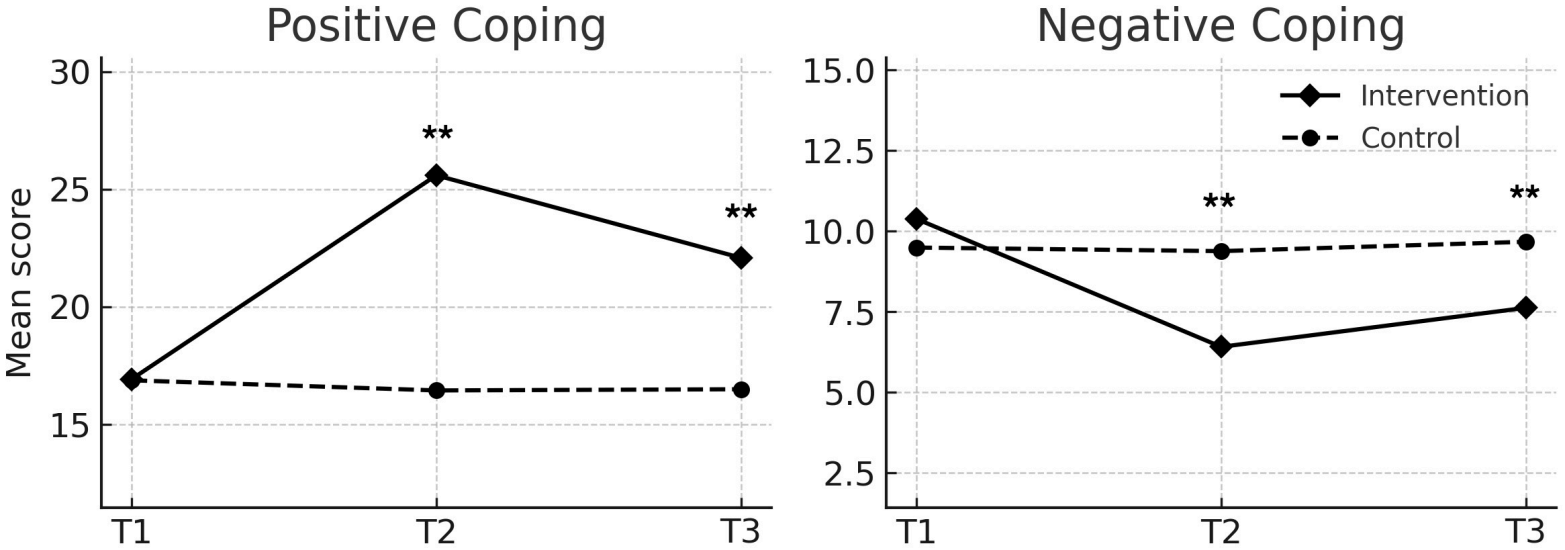
Changes in positive and negative coping scores over time in the intervention and control groups. Values are presented as means. T1 indicates baseline, T2 indicates post-intervention, and T3 indicates 3 month follow-up. Repeated-measures ANOVA showed significant effects of group, time, and group × time interaction for both positive coping (*p* < 0.001) and negative coping (*p* < 0.05). ^**^ indicates *p* < 0.01 for between-group comparisons at the same time point.

For positive coping, the intervention group showed significant increases at T2 (*M* = 25.59, *SD* = 5.29) and T3 (*M* = 22.08, *SD* = 5.83) relative to the control group (T2: *M* = 16.44, *SD* = 4.25; T3: *M* = 16.49, *SD* = 4.19; both *P* < 0.001). For negative coping, the intervention group showed a significant decrease at T2 (*M* = 6.41, *SD* = 3.08) and T3 (*M* = 7.62, *SD* = 2.62) compared to the control group (T2: *M* = 9.38, *SD* = 2.39; T3: *M* = 9.67, *SD* = 2.60; both *P* < 0.001) (Figure 4).

#### Anxiety and depression

**Figure 5:**
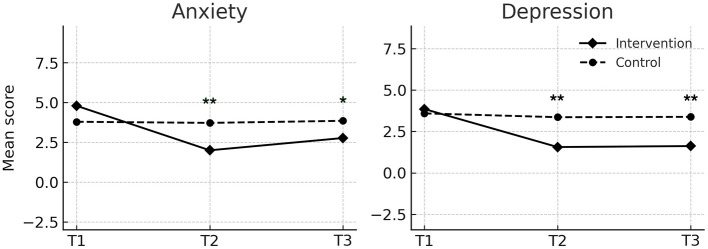
Changes in anxiety and depression scores over time in the intervention and control groups. Values are presented as means. T1 indicates baseline, T2 indicates post-intervention, and T3 indicates 3-month follow-up. Repeated-measures ANOVA showed significant effects of group, time, and group × time interaction for both outcomes (all *p* < 0.001). ^*^ and ^**^ indicate between-group differences at p < 0.05 and p < 0.001, respectively.

Scores for anxiety and depression significantly decreased in the intervention group at both T2 and T3, while remaining stable in the control group. At T2, anxiety scores in the intervention group (*M* = 2.00, *SD* = 1.62) were significantly lower than those in the control group (*M* = 3.72, *SD* = 1.99, *P* < 0.001), with similar group differences at T3 (*P* = 0.013). Depression scores also decreased significantly in the intervention group compared to the control group at both time points (*P* < 0.001) (Figure 5).

#### Physiological indicators

No statistically significant differences were observed in heart rate, systolic blood pressure, or diastolic blood pressure between the intervention and control groups at any assessment point (*P* > 0.05).

Additional analyses based on estimated marginal means are provided in Supplementary figure 1.

## Discussion

This study provides preliminary evidence that a forgiveness-based intervention program may offer a new avenue for developing PTG-focused psychological interventions in the MHD population.

Preliminary findings demonstrated that the forgiveness intervention program significantly enhanced PTG and forgiveness scores in the intervention group compared to the control group, with these improvements sustained during the 3 month follow-up. These findings are consistent with previous research demonstrating the effectiveness of forgiveness-based interventions in fostering positive psychological change following traumatic experiences (Cui et al., 2017; Ha et al., 2019; Lopez et al., 2021; Skalski-Bednarz et al., 2024). For example, Ha et al. (2019). reported that forgiveness interventions helped reduce trauma-related symptoms, alleviate feelings of shame and depression, and promote PTG among individuals exposed to trauma. In the present study, both groups exhibited similarly low PTG levels at baseline, supporting the validity of post-intervention comparisons. While conventional health education may improve patients‘ understanding of their illness, it often lacks emotional depth and does not sufficiently address motivational factors (Jiakponna et al., 2024). In contrast, the forgiveness-based intervention program used in this study incorporated motivational interviewing, cognitive reframing, and perspective-taking activities specifically tailored to the experiences of patients receiving maintenance hemodialysis (Natale et al., 2019). Participants were encouraged to revisit their illness narratives, express related emotions, and engage in constructive cognitive restructuring. Activities such as reflective journaling, guided imagery, and gratitude exercises provided opportunities for patients to reinterpret their illness experiences in a more positive light, fostering inner strength and a deeper appreciation of life (Elliott et al., 2015). Furthermore, interactive components that cultivated empathy and strengthened interpersonal relationships supported patients' relational growth, which is a central dimension of PTG (Ye et al., 2025). These integrated strategies contributed to the observed improvements in PTG and highlight the added value of structured forgiveness-based interventions compared to routine health education for patients undergoing maintenance hemodialysis.

In this study, both groups exhibited similar forgiveness levels at baseline; however, only the intervention group showed significant improvements post-intervention and at the 3 month follow-up, demonstrating the effectiveness of the forgiveness-based program. Forgiveness is commonly referred to as a positive psychological process in which individuals intentionally reshape maladaptive thoughts, emotions, and behaviors in response to distressing experiences (Enright et al., 1998). This finding aligns with cognitive-behavioral theory, which emphasizes that lasting emotional change requires the restructuring of dysfunctional beliefs (Beidel and Turner, 1986). The intervention facilitated this process through techniques such as the ABC model of emotion and guided interpretation of ambiguous images, helping patients identify and reframe irrational thought patterns. In addition, discussions on the meaning of forgiveness, perceived illness-related gains, and personal experiences fostered trust and engagement, while face-to-face instruction and skill-based exercises strengthened emotional competence and self-efficacy. These components jointly contributed to the sustained improvements in forgiveness among participants. By contrast, (Hansen et al. (2009) reported that standard clinical care lacking an explicit focus on forgiveness failed to enhance forgiveness outcomes in patients with chronic illness, underscoring the added value of structured forgiveness-based interventions for patients receiving maintenance hemodialysis.

The intervention produced significant improvements in coping strategies. Participants in the intervention group reported increased use of positive coping and reduced reliance on negative coping both post-intervention and at 3 month follow-up (*P* < 0.001). No significant changes were observed in the control group. These findings support evidence that enhancing positive psychological resources—such as forgiveness and PTG—can improve adaptive coping (Auriemma et al., 2022). Forgiveness, as a cognitive-emotional shift from negative to positive perspectives, may have encouraged patients to reappraise their illness constructively rather than avoid or surrender. Through reflective activities, cognitive restructuring, and forgiveness meditation, patients developed greater insight into their illness, replacing maladaptive responses with more engaged coping behaviors (Hsu, 2021). Educational components warning against negative coping and offering actionable alternatives further reinforced this shift. Overall, the forgiveness-based intervention proved more effective than routine care in reducing maladaptive coping behaviors and reinforcing positive coping in patients undergoing maintenance hemodialysis.

The forgiveness-based intervention significantly alleviated anxiety and depression among MHD patients. Compared to the control group, the intervention group reported markedly lower anxiety scores both immediately after the intervention (*P* < 0.001) and at the 3 month follow-up (*P* = 0.013), along with significant reductions in depression at both time points (*P* < 0.001). These findings are consistent with previous research showing that MHD patients often experience high levels of emotional distress due to persistent symptom burden and lifestyle disruptions (Chatrung et al., 2015). The intervention incorporated techniques such as motivational interviewing, emotional expression, guided imagery, and progressive muscle relaxation (Alhawatmeh et al., 2022; Wu et al., 2025), which provided practical strategies for emotional regulation. By helping MHD patients manage psychological stressors more effectively, the intervention contributed to the reduction of anxiety and depression.

However, physiological indicators including heart rate and blood pressure showed no statistically significant differences between groups across assessment points (all *P* > 0.05). The minor fluctuations observed suggest that short-term psychological interventions may have limited effects on physiological parameters among MHD patients, whose cardiovascular status often involves complex, multifactorial challenges. May et al. (2014) reported that forgiveness-based interventions significantly alleviated myocardial ischemia induced by anger in patients with coronary artery disease, which contrasts with the present findings. Future studies with extended intervention durations and follow-up periods may be needed to better evaluate potential physiological benefits in this population.

## Conclusions

This study demonstrated that a forgiveness-based psychological intervention effectively enhanced posttraumatic growth and forgiveness, promoted adaptive coping, and alleviated anxiety and depression among MHD patients. In contrast, no significant changes were observed in physiological outcomes such as heart rate and blood pressure. These findings suggest that forgiveness-focused interventions can improve key psychological outcomes in hemodialysis care, offering a feasible and low-cost approach with potential for broader application in clinical practice.

## Limitations

Our study has some limitations. The primary limitation was that it was conducted at a single center, which may limit the generalizability of the findings to other settings or populations. Another limitation was the inability to randomly assign patients to the intervention and control groups, which may have introduced imbalances in participant characteristics. Future studies should consider adopting randomized, multicenter designs to improve the representativeness and robustness of the results.

## Data Availability

The original contributions presented in the study are included in the article/Supplementary material, further inquiries can be directed to the corresponding author.
