# Supplementary material for: Effectiveness of a forgiveness-based intervention to promote post-traumatic growth in hemodialysis patients: an experimental controlled study
Source: Front Psychol. 2025 Oct 20;16:1680748. doi: 10.3389/fpsyg.2025.1680748 (PMC12592882; doi:10.3389/fpsyg.2025.1680748)
Supplement: Supplementary file 1 [file Image_1.DOCX]

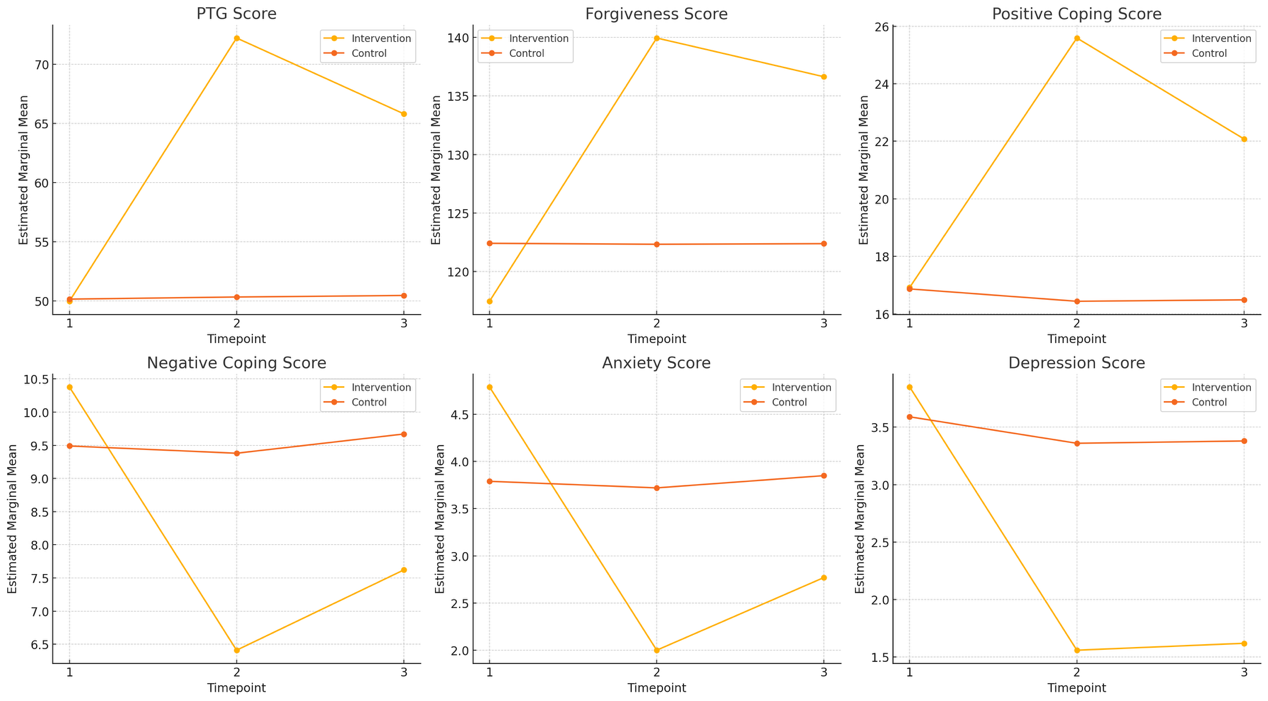


FIGURE 1

Estimated marginal means of key psychological outcomes at three time points (1 = pre-intervention, 2 = post-intervention, 3 = 3-month follow-up) for the intervention and control groups. Outcomes include posttraumatic growth (PTG), forgiveness, positive coping, negative coping, anxiety, and depression.
